# Unlocking the potential of computer vision in precision pig farming: a call for a collaborative data and AI models platform

**DOI:** 10.1093/af/vfag003

**Published:** 2026-01-30

**Authors:** Hassan-Roland Nasser, Claudia Kasper

**Affiliations:** Agroscope, Digital Production Group. Tanikon 1, Ettenhausen, 8356, Switzerland; Agroscope, Animal GenoPhenomics Group, Posieux, 1752, Switzerland

**Keywords:** computer vision, data repository, large vision models, livestock farming, model repository

Implications

- Lowering entry barriers for research: Through coordinated engagement of researchers and funding bodies, the proposed collaborative platform lowers entry barriers by providing standardized datasets, benchmarked models, and reproducible pipelines. This facilitates its adoption by pig scientists while offering the AI community a domain-specific testbed for developing and validating vision-centric models tailored to pigs.
- Enabling reproducible and cumulative scientific progress: collaborative data contributions and standardized modeling frameworks allow training on large, multimodal datasets. This supports reproducibility and comparability, fosters cumulative progress, and paves the way for robust, pig-specific foundation models.
- Providing a scalable and transferable blueprint: Although focused on pigs, the proposed framework is transferable to other species facing similar challenges related to data fragmentation, annotation costs, and model generalizability.

## Computer Vision is Strategic for Precision Pig Farming

Computer vision (CV) is increasingly used in precision pig farming (PPF) and pig research fields, such as welfare, nutrition and genetics, as it enables noninvasive, continuous, and objective monitoring of animals in both research and commercial farms. By extracting quantitative information from visual data streams, CV provides a wide range of indicators related to welfare, health, productivity, and management indicators; parameters that are central to sustainable pig production. These include, for example, the automated detection of behaviors and conditions relevant to welfare and husbandry such as aggression, tail biting, sickness, or farrowing-related events (Fraser et al., 2023; D. Liu et al., 2020), as well as the estimation of production and sustainability-related traits such as body weight, body condition, and feed efficiency (da Cunha et al., 2024; Dong et al., 2025). Such data could be then also used in breeding programs. Recent literature reviews provide a detailed and comprehensive overview about the use of CV for precision livestock farming (Y. Wang et al., 2023; Menezes et al., 2024; Rohan et al., 2024; Scott et al., 2024; Assimakopoulos et al., 2025; Brassó et al., 2025; Marchegiani et al., 2025; Michelena et al., 2025).

Further, CV facilitates early detection of health and welfare issues, thereby reducing suffering and production losses as well as improving resource efficiency (Kashiha et al., 2013; Menezes et al., 2024). Additionally, it provides tools to demonstrate compliance and accountability with societal expectations regarding animal welfare, and environmental sustainability. In this respect, CV provides quantitative observations that can complement existing management and monitoring practices.

Furthermore, CV complements and extends existing approaches within PPF. Unlike sensor-based methods that often require invasive attachment to the animal, CV provides scalable, contactless measurements applicable at both individual and group levels. This makes it particularly well-suited for intensive pig farming systems, where high animal densities necessitate extensive monitoring, and continuous, automated observation can assist human caretakers in maintaining oversight.

Importantly, the strategic value of CV is further amplified when visual information is integrated with complementary data modalities within a unified framework, for example, as outlined in the “phenome” of Pérez-Enciso and Steibel (2021). Vision-derived phenotypes provide noninvasive, real-time information on pigs. This can serve as an anchor, allowing precise alignment of heterogeneous data streams through shared timestamps, such as production (growth, feed intake and efficiency), environmental (temperature and humidity), physiological, multiomics and potentially even genomic data. Rather than replacing existing data sources (e.g., accelerometers and RFID, environmental sensors, or farm management data), vision-centric multimodal integration enriches visual data with contextual information, enabling more robust and generalizable inference. This approach underpins the vision-centric multimodal platform proposed below, where CV serves as the central modality for integrating other kinds of data. Taken together, the noninvasive, real-time, high-resolution attributes of CV explain its growing importance in pig production research.

Despite rapid advances in field-agnostic CV, the development of CV methods tailored to pigs remains limited.

The disparity of advances in field-agnostic CV and its application in PPF is summarized in Table 1.

**Table 1.** Comparative overview of mainstream computer vision research and computer vision applied to PPF. The first column lists comparison dimensions; the second column summarizes characteristics of field-agnostic computer vision; the third column summarizes characteristics specific to computer vision in PPF. We highlight the differences in research pace, data availability, methodological accessibility, challenges, scalability, and overall trajectory.

Dimension	Field-agnostic computer vision	Computer vision in PPF
*Research pace*	Rapid progress driven by foundation models, multimodal learning, and large-scale benchmarks.	Slow progress with fragmented, small-scale studies.
*Data resources*	Large, standardized, and open datasets (e.g., ImageNet, COCO, OpenLLaVA datasets).	Lack of open data; small data sets with limited standardization for annotation and metadata.
*Methodological accessibility*	Widely available reproducible pipelines, pretrained models, and open-source frameworks.	Many solutions remain inaccessible due to lack of reproducible code, expertise gaps, and computational barriers.
*Challenges*	Generic (occlusion, lighting, generalizability).	Domain-specific (crowded pens, uniform coloration, complex social behaviors).
*Scalability and deployment*	Increasingly deployed across industries (autonomous driving, healthcare, robotics).	Mostly pilot studies; limited integration into real-world farm infrastructure.
*Trajectory*	Accelerating adoption and innovation.	Risk of stagnation without systemic changes.

Several factors contribute to this stagnation. Some challenges are common across CV domains, including occlusion, variable lighting conditions, and limited model generalizability (Chen et al., 2020; D. Liu et al., 2020; M. Wang et al., 2023). Others are specific to pig farming, such as the difficulty of tracking individuals within crowded pens (Parmiggiani et al., 2023; Devi et al., 2024; Luo et al., 2025), the visual homogeneity of pig body coloration (D. Liu et al., 2025), which provides few discriminative features and the context-dependent nature of social and aggressive behaviors (Kim et al., 2024; Lee et al., 2024). Although technical solutions to some of these problems have been proposed (e.g., improved multiobject tracking for occlusion, data augmentation for lighting variability), these methods remain inaccessible to many researchers due to computational and infrastructural constraints, as well as the limited availability of reproducible code and pipelines and pretrained models. Importantly, many of these challenges are not unique to PPF, suggesting that they alone cannot explain the markedly slower research pace observed in PPF.

### Bottleneck 1: Data fragmentation and annotation inefficiency

In addition to challenges previously mentioned, one recurring limitation concerns data availability and annotation practices. Research groups frequently annotate their own limited datasets (Fraser et al., 2023; Ji et al., 2023), often without leveraging or harmonizing existing data resources. The absence of standardized, well-documented, open-access datasets and benchmarks restricts reproducibility and slows the transfer of methods and models across contexts. Consequently, studies are often difficult to replicate or extend across farm contexts, and their findings are rarely scalable to other farm settings or deployable within operational farm infrastructure. This fragmented data landscape, reflected in the lack of shared benchmarks and harmonized annotation practices (Table 1), further limits reproducibility and cross-study comparability.

### Bottleneck 2: Limited model reproducibility and generalizability

Beyond data-related constraints, progress in PPF is further limited by the difficulty of reproducing, scaling, and transferring models across farms and contexts. Meanwhile, the broader CV field has rapidly adopted large-scale benchmarks, driven by competition and breakthroughs in areas such as foundation models, multimodal learning, and self-supervised representation learning. Similar paradigm shifts have recently been highlighted as both necessary and challenging for agriculture and livestock applications, where data heterogeneity, annotation costs, and deployment constraints limit direct adoption of general-purpose models (Radford et al., 2021; Bommasani et al., 2022; Li et al., 2024; Nedungadi et al., 2025). Researchers working at the intersection of CV and PLP struggle to keep pace with these developments due to constraints in data availability, annotation cost, computational resources, and reusable benchmarking infrastructure, which reinforces the divergence in research trajectories highlighted in Table 1 and leads to missed opportunities to adapt state-of-the-art methodologies to domain-specific problems in pig production. These two bottlenecks motivate the conceptual framework outlined in the following sections: a collaborative data initiative to address fragmentation and the inefficiency and lack of standardization in annotation, and a shared model platform to improve reproducibility, scalability, and generalizability.

As a result, while proof-of-concept applications continue to emerge, their broader impact remains constrained by the limitations mentioned above. Addressing these structural limitations requires a shift from isolated case studies toward systemic solutions that leverage the potential of small data sets created within a research context to improve scalability and predictive capacity.

## Fighting the Data Gap: Toward an International Collaborative Initiative

Most current studies investigating the application of CV to pig production follow a traditional model-development pathway in which each research group independently collects and annotates images, resulting in the above-mentioned scarcity of high-quality, standardized datasets. Manual annotation, which requires extensive training and domain expertise, remains one of the most time-intensive and resource-demanding tasks in CV, consuming valuable researcher and student time that could otherwise be invested in methodological innovation or interdisciplinary integration.

While a few publicly available datasets exist (Pan et al., 2023; T. Liu et al., 2025; Wutke et al., 2025), they are often fragmented across repositories, inconsistent in format, and poorly documented. Researchers seeking to build upon these published resources must therefore dedicate significant effort to searching, downloading, re-annotating, and standardizing data; a process that introduces inefficiencies and limits reproducibility. The absence of a comprehensive overview and repository for available datasets further exacerbates this issue, leaving the community without a clear picture of what data exist and how they may be reused.

Addressing this data gap requires coordination beyond individual research groups: Instead of each group annotating data solely for its own use, annotations could be shared across the community. A centralized repository that integrates both publicly available and newly contributed datasets that are harmonized, standardized, and accompanied by clear and comprehensive metadata, would substantially increase the return on investment for annotation efforts. Such a platform could support multiple CV modalities relevant to PPF (e.g., classification, detection, keypoint estimation, segmentation, tracking, and re-identification) while capturing variability across farms, camera systems, and environmental conditions.

Shared annotation efforts may reduce redundant work and improve cross-study comparability. Over time, standardized datasets may facilitate broader reuse of methods developed within the CV community. On the long term, the available assets (data, models, infrastructure, and codes) can potentially attract the theoretical AI community to develop multimodal models tailored for pigs.

## Towards a Large Vision Model for Pigs via a Collaborative Platform

**Figure 1.**
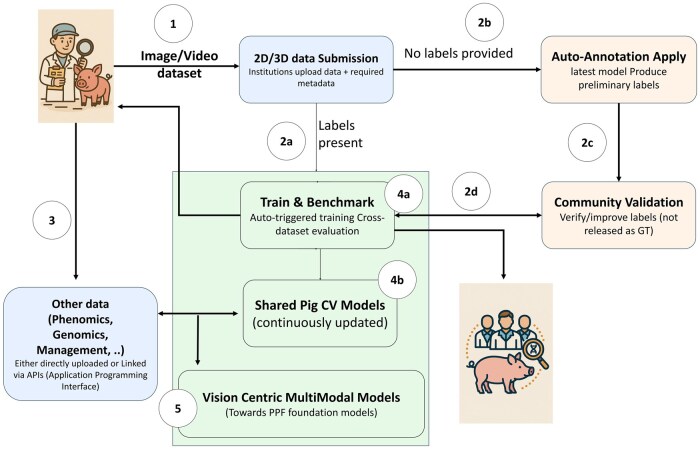
Return on investment and workflow of the proposed open data and multimodal platform. Institutions or researchers contribute image datasets (1) that can be either annotated (2a) or unannotated (2 b). (3) Data sets can be directly linked to other phenomics or genomics resources (either directly uploaded or fetched from external repositories). Annotated image datasets directly trigger new training cycles (4a), generating updated models that are benchmarked against all existing datasets and subsequently released to the community (2b). Unannotated image datasets are processed through inference routines to produce preliminary labels, which are then refined through community validation (2c). Once validated, these datasets are incorporated as annotated data, thereby initiating new training and benchmarking cycles (4a). The diagram further illustrates the return-on-investment principle: a single dataset contribution yields access to multiple datasets, trained models, and benchmarking resources for participating researchers. Ultimately, researchers contributing phenomics datasets in addition to visual datasets (5) would enable the development of multimodal AI models that link visual phenotypes to underlying biological and management processes, allowing visual observations to be interpreted in relation to genetics, physiology, and production context.

This section outlines a conceptual framework for collaborative model development rather than prescribing specific architectures or infrastructure designs. Building upon the proposed initiative for collaborative dataset sharing and annotation, a natural next step is the development of a centralized platform that not only hosts data but also supports the creation and continuous refinement of large-scale pig-specific CV models (Figure 1). Such a platform would provide guidance for data/image acquisition and image/video annotation, integrate new datasets contributed by the community and automatically update model parameters, enabling the generation of increasingly robust and generalizable models over time.

Although establishing this platform requires additional effort from the scientific community, integrating it as both a data repository and a model engine would yield significant efficiency gains compared to developing these initiatives independently. By coupling data harmonization with collaborative model development, the platform could become a cornerstone for reproducible and scalable applications of CV in pig production. While a detailed technical specification is beyond the scope of this article, two key resources are essential for the establishment and long-term viability of such an infrastructure, which require reliable funding:

1. Human resources: A minimum team of one full-stack web developer to design and maintain the platform infrastructure, one CV engineer to implement training and evaluation pipelines, and one platform manager serving as a primary point of contact for communication, community building, and representing the strategic interest of this research community.
2. Computational resources: a cloud-based hosting environment with dedicated budget for secure data storage, model training, and evaluation routines.

We propose the following workflow for the platform:

1. Dataset submission: Institutions contribute datasets following standardized guidelines, including required metadata.
2. Annotated datasets: When a dataset with validated annotations is added, the platform automatically triggers training, producing a new model checkpoint. This checkpoint is (a) made publicly available for immediate use and (b) systematically evaluated against all existing datasets to assess generalizability. Unannotated datasets: When unannotated datasets are submitted, the current model is used to generate preliminary labels that serve only as annotation support. To limit the risk of bias amplification and feedback-loop effects, these labels must be validated by qualified human annotators and benchmarked against independently annotated datasets before being reused for training.
3. This mechanism establishes a virtuous cycle in which the quality and quantity of data continuously enhance model performance, while model updates facilitate more efficient annotation and accelerate research progress. By providing benchmarked models and shared evaluation procedures, the platform is intended to address reproducibility and scalability limitations identified in current CV applications in PPF.

Moreover, continual model retraining entails nontrivial computational and financial costs, suggesting that model updates would need to follow controlled and periodic schedules rather than continuous retraining.

### From vision to vision-centric multimodal AI platform for PPF

While this article focuses primarily on CV, the proposed platform naturally lends itself to a broader, vision-centric multimodal AI framework for PPF. In practice, such a platform could host and align multiple data modalities collected at the animal or group level, with vision acting as a central, noninvasive anchor modality. Visual data are often tightly coupled to phenotypic traits that reflect behavior, health status, and physiological processes, thereby providing a bridge between external observations and internal biological states. By integrating complementary information such as blood and biomarker measurements, production data, and health records, complex phenotypes (e.g., nutrient efficiency), and multiomics data (e.g., transcriptomics, DNA methylation, proteomics, metabolomics) for the same individuals, we can create richer, more comprehensive representations of individual pigs and production systems. In the future, the possibility of integration with genomic data repositories, such as the European Nucleotide Archive or the National Center for Biotechnology Information, could be explored, which host a wealth of genomics and other omics data submitted by the research community. This would open up interesting possibilities for genome-to-phenome research and facilitate the development of genomic prediction models for future practical applications. While a detailed treatment of multimodal architectures lies beyond the scope of this article, we highlight this direction as a natural extension of the proposed platform. Such multimodal integration has the potential to improve the biological validity and generalizability of models across contexts, and ultimately facilitate the development of smarter digital twins for pigs that link visual phenotypes to underlying biological and management processes. Nevertheless, these largely data-driven approaches improve validity but offer limited mechanistic insight.

### Implementation challenges and mitigation strategies

While the need for an international data collaborative platform is clear, its implementation faces substantial logistical and governance challenges. Barriers to data sharing in PPF and research are not purely technical: institutions and companies may be reluctant to contribute valuable or proprietary data due to concerns over intellectual property, competitive advantage and legal liability. Moreover, the costs of data annotation, standardization, pseudonymization, and documentation are substantial. Beyond financial aspects, there is also a lack of established guidelines and training for these processes. Participation therefore cannot rely solely on goodwill.

A viable collaborative model must instead be built around clear incentives. These may include preferential access to aggregated datasets, shared annotation efforts, benchmark results, pretrained models, and visibility or co-authorship for contributing researchers and institutions. From a governance perspective, tiered access models can help mitigate intellectual property concerns by allowing contributors to define usage conditions ranging from fully open to controlled or embargoed access. Technical mitigation strategies, such as standardized metadata schemas, automated pseudonymization pipelines, and centralized preprocessing workflows, can further reduce the burden placed on individual contributors while improving interoperability and reproducibility.

Such approaches have already proven successful in other data-intensive scientific fields. In livestock sciences in particular, initiatives like FAANG (Harrison et al., 2021) as well as AG2PI, and more general, the USDA Blueprint for Animal Genome Research 2018–2027 (Rexroad et al., 2019), demonstrate the effectiveness of combining governance structures with incentive mechanisms. An ongoing EU COST action (EU-LI-PHE; CA22112) offers a platform to advance these ideas further. Additionally, efforts to establishing long-term frameworks, such as the Genophenix European Research Infrastructure and the Elixir Focus Group Domestic Animals Genome and Phenome, highlight the potential for sustainable collaboration. These examples not only show that such challenges, can be overcome, but also provide opportunities for coordination and collaboration with the initiative proposed in this paper, or even for integration into these existing efforts.

## Conclusion

We presented a call for collective action to establish a collaborative data and model platform aimed at accelerating progress in pig research and farming through CV. This initiative explicitly recognizes the need to address data governance and computational constraints to ensure its sustainability. While this initiative focuses on pigs, its underlying principles and the proposed infrastructure are broadly applicable to other livestock species, where CV can enhance welfare, efficiency, and sustainability, as well as to plant sciences, which face comparable challenges of fragmented datasets, image variability, and limited model generalizability. By fostering a collaborative community and actively seeking long-term funding to provide shared resources and scalable solutions, this initiative aims to establish a sustainable, vision-centric foundation for future multimodal integration, not only for pig research, but as a transferable model for other livestock and agricultural sciences facing similar data and reproducibility challenges.

## Author Biographies

**Claudia Kasper** investigates the genetics of sustainability traits in pigs and cattle as a researcher in the Animal GenoPhenomics research group at Agroscope. She focuses on feed and nutrient efficiency, particularly nitrogen use efficiency and methane emissions, as well as birth weight variability. She is passionate about advancing phenotyping methods for novel livestock traits and is a strong advocate for data sharing and reuse to accelerate scientific progress. Before taking up her current position, she completed her PhD in Evolutionary Genetics at the University of Bern.

**Hassan-Roland Nasser** is a researcher at Agroscope in the Digital Production group. His work focuses on applying artificial intelligence to challenges in agriculture, particularly in livestock and smart farming. Before joining Agroscope, he lectured in data science at French and Lebanese universities. He holds a PhD in Computational Neuroscience from INRIA and a degree in Biomedical Engineering from the University of Technology of Compiègne. His research interests include computer vision and its applications for automating agricultural observation and image analysis.
